# Review of preclinical data of PF-07304814 and its active metabolite derivatives against SARS-CoV-2 infection

**DOI:** 10.3389/fphar.2022.1035969

**Published:** 2022-11-11

**Authors:** Wujun Chen, Yingchun Shao, Xiaojin Peng, Bing Liang, Jiazhen Xu, Dongming Xing

**Affiliations:** ^1^ Cancer Institute, The Affiliated Hospital of Qingdao University, Qingdao University, Qingdao Cancer Institute, Qingdao, China; ^2^ School of Life Sciences, Tsinghua University, Beijing, China

**Keywords:** PF-07304814, pharmacology, pharmacokinetics, toxicology, derivatives, SARS-CoV-2

## Abstract

Main protease (M^pro^) is a superior target for anti-SARS-COV-2 drugs. PF-07304814 is a phosphate ester prodrug of PF-00835231 that is rapidly metabolized into the active metabolite PF-00835231 by alkaline phosphatase (ALP) and then suppresses SARS-CoV-2 replication by inhibiting M^pro^. PF-07304814 increased the bioavailability of PF-00835231 by enhancing plasma protein binding (PPB). P-glycoprotein (P-gp) inhibitors and cytochrome P450 3A (CYP3A) inhibitors increased the efficacy of PF-00835231 by suppressing its efflux from target cells and metabolism, respectively. The life cycle of SARS-CoV-2 is approximately 4 h. The mechanisms and efficacy outcomes of PF-00835231 occur simultaneously. PF-00835231 can inhibit not only cell infection (such as Vero E6, 293T, Huh-7.5, HeLa^+angiotensin-converting enzyme 2 (ACE2)^, A549^+ACE2^, and MRC-5) but also the human respiratory epithelial organ model and animal model infection. PF-07304814 exhibits a short terminal elimination half-life and is cleared primarily through renal elimination. There were no significant adverse effects of PF-07304814 administration in rats. Therefore, PF-07304814 exhibits good tolerability, pharmacology, pharmacodynamics, pharmacokinetics, and safety in preclinical trials. However, the Phase 1 data of PF-07304814 were not released. The Phase 2/3 trial of PF-07304814 was also suspended. Interestingly, the antiviral activities of PF-00835231 derivatives (compounds 5–22) are higher than, similar to, or slightly weaker than those of PF-00835231. In particular, compound 22 exhibited the highest potency and had good safety and stability. However, the low solubility of compound 22 limits its clinical application. Prodrugs, nanotechnology and salt form drugs may solve this problem. In this review, we focus on the preclinical data of PF-07304814 and its active metabolite derivatives to hopefully provide knowledge for researchers to study SARS-CoV-2 infection.

## 1 Introduction

Coronavirus disease 2019 (COVID-19), which is caused by severe acute respiratory syndrome coronavirus-2 (SARS-CoV-2), quickly spread worldwide since its first discovery on 5 December 2019. To date, COVID-19 still impacts us all. Multiple pharmaceutical interventions and vaccines have been approved to mitigate or prevent the course of this disease. The drugs interventions include Paxlovid (nirmatrelvir (PF-07321332)/ritonavir), Lagevrio (molnupiravir), Veklury (remdesivir), Kineret (anakinra), Regkirona (regdanvimab), RoActemra (tocilizumab), Ronapreve (casirivimab/imdevimab), Xevudy (sotrovimab), BRII-196/BRII-198, etesevimab/bamlanivimab, bamlanivimab, Regkirona, and Ronapreve, and the vaccines include Comirnaty, BNT162b2, Spikevax, CoronaVac, and BBIBP-CorV (Sokullu E, 2021; Fernandes Q, 2022). However, these interventions have not been able to completely stop the risk of COVID-19 infection, and more drugs still need to be developed to overcome the outbreak.

The main protease (M^pro^) of SARS-CoV-2 (also named 3C-like protease, 3CL^pro^ or nsp5) plays a key role in viral replication. Interestingly, M^pro^ is highly conserved in coronaviruses and is not a human homolog, suggesting that M^pro^ is an important target for antiviral drug development**.** PF-07304814 (also named lufotrelvir), which is a highly soluble phosphate prodrug of PF-00835231, was the first M^pro^ inhibitor to enter clinical trials. In fact, the oral form of PF-07304814, named nirmatrelvir, was developed and approved by the U.S. Food and Drug Administration (FDA) on 2021.12.22 (Simsek-Yavuz and Komsuoglu Celikyurt, 2021). However, PF-07304814 and nirmatrelvir are different not only in dosage form but also in structure. In this review, we focus on the preclinical data of PF-07304814 and its active metabolite derivatives to hopefully provide knowledge for researchers to study SARS-CoV-2 infection.

## 2 M^pro^: A superior target for anti-SARS-COV-2 drugs

The biological structure of SARS-CoV-2 contains an RNA gene chain, nonstructural proteins (nsps), structural proteins and accessory proteins. There are 16 nsps, from nsp1 to nsp16; 4 structural proteins, including spike (S) protein, membrane (M) protein, envelope (E) protein, and nucleocapsid (N) protein; and 9 accessory proteins, including ORF3a, ORF3b, ORF6, ORF7a, ORF7b, ORF8, ORF9b, ORF9c, and ORF10. Accessory proteins are not necessary for virus replication but can help the virus fight host viral defense mechanisms (Kim et al., 2020; Yao et al., 2020). M^pro^ is a cysteine protease with a distinct substrate preference for glutamine at the site Leu-Gln/(Ser, Ala, Gly). The replication of SARS-CoV-2 depends on polyproteins, and M^pro^ dominates the replication of the Coronaviridae family of viruses by cleaving polyproteins when the viral RNA enters the host cell. Polyproteins, including pp1a and pp1ab, are encoded by two-thirds of the 5’ end of the coronavirus viral genome. These polypeptides are cleaved into mature nsps *via* two viral cysteine proteases, M^pro^ and papain-like protease (PL^pro^). These nsps promote viral replication by assembling into a replication-transcription complex (Zhang L, 2020).

M^pro^ of SARS-CoV-2 shows >96% sequence identity with M^pro^ of SARS-CoV; in particular, the M^pro^ binding pocket residues are strongly conserved. Most importantly, mammals, including humans, mice, rats, pigs, and monkeys, lack homologs of M^pro^, and mammalian proteases do not recognize the M^pro^ sequence. Deliberately developing highly selective M^pro^ inhibitors may reduce the risk of side effects from taking these drugs. Indeed, a large number of compounds have been found to exhibit inhibitory activity against M^pro^; however, only nirmatrelvir has been approved (Garcia-Lledo et al., 2021). Nirmatrelvir may cause a second round of COVID-19 symptoms, embryonic developmental toxicity and changes in host gene expression (Dai et al., 2022). Therefore, there is an urgent need for effective and bioavailable antiviral drugs to treat SARS-COV-2 infection.

## 3 The mechanism of PF-07304814 against SARS-CoV-2 infection

### 3.1 The binding sites of PF-00835231 and M^pro^

**FIGURE 1:**
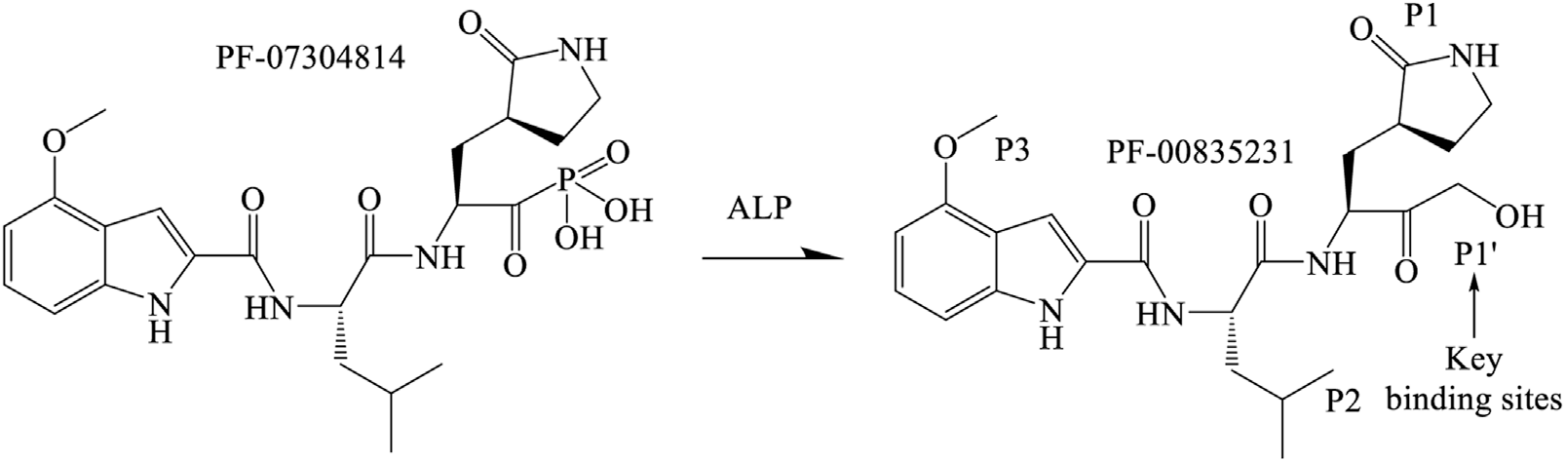
Mechanism and major binding site of PF-07304814 to suppress M^pro^ activity.

The hydroxymethyl ketone of PF-00835231 forms a covalent bond with the M^pro^ active site cysteine (Cys145) to suppress M^pro^ activity (Figure 1) (Giacomo G. Rossetti, 2020; Hangchen Hu, 2022). The P1’ position of PF-00835231 has a hydroxymethyl group that resembles a serine residue, that enables M^pro^ to easily bind to this moiety. Specifically, the groups in the P1 position of PF-00835231 establish hydrogen bonds with M^pro^ residues such as His163, Glu166 and Phe140. The isobutyl hydrocarbon group at the PF-00835231 P2 position forms stacking interactions with the His41 imidazole ring as well as other nearby residues, such as His164, Met165 and Gln189. The PF-00835231 P3 position is exposed to the solvent and stabilized by hydrogen bonding with the backbone atoms of Met165, Glu166 and Glu189 (Baig et al., 2021; Ramos-Guzman et al., 2021).

### 3.2 PF-07304814 was metabolized into the active compound PF-00835231 by alkaline phosphatase

The active compound PF-00835231 suppresses coronavirus replication. However, PF-00835231 has limited clinical application due to its poor solubility and bioavailability. ALP could increase the exposure of phosphate ester prodrugs to their respective active metabolites. Many phosphate ester prodrugs have also been approved by the FDA, suggesting that designing the phosphate ester prodrug of PF-00835231 could increase its aqueous solubility. PF-07304814 is a phosphate ester prodrug of PF-00835231 that is rapidly metabolized into PF-00835231 by ALP, which then suppresses SARS-CoV-2 infection (Fulmali A, 2022).

### 3.3 PF-07304814 increased the bioavailability of PF-00835231 by enhancing plasma protein binding

The total drug concentration in plasma consists of two parts, the free plasma drug concentration (UPC) and the bound plasma protein concentration. PPB [also called the binding rate of plasma protein (BRPP)] is a dynamic process that acts as a drug warehouse. Drugs with high PPB are eliminated slowly *in vivo*, as they remain in the body for a long time and have stable efficacy, suggesting that PPB plays a key role in the effects of a drug and its length of maintenance (Charlier et al., 2021). PF-07304814 exhibits high PPB in humans (81.6%), monkeys (63.9%), dogs (68.8%), and rats (62.1%), while the PPB of its metabolite PF-00835231 in these species are 55.1%, 55.9%, 58.4%, and 67.3%, respectively, suggesting that PF-07304814 has a higher PPB than its metabolite PF-00835231 *in vivo* (Boras et al., 2021). Therefore, PF-07304814 increased the bioavailability of PF-00835231 by enhancing plasma protein binding (PPB) as a drug warehouse.

### 3.4 The efficacy of PF-00835231 to suppress M^pro^ activity

PF-00835231 can tightly and specifically bind to SARS-CoV-2 M^pro^ with half-maximal inhibitory concentration (IC_50_) values ranging from 0.27 nM to 8 nM (Hoffman et al., 2020; Boras et al., 2021; Vandyck et al., 2021; Johansen-Leete et al., 2022). PF-00835231 also suppressed M^pro^ of other coronaviruses with inhibition constants (Ki values) ranging from 30 p.m. to 4 nM, such as alpha-CoVs (including NL63-CoV, HCoV-229E, PEDV, and FIPV), beta-CoVs (including HKU4-CoV, HKU5-CoV, HKU9-CoV, MHV-CoV, MERS-CoV, OC43-CoV, HKU1-CoV), and gamma-CoVs (including IBV-CoV). In addition, the affinity of PF-00835231 to Mpro is independent of pH, suggesting that PF-00835231 exhibits very stable binding to and inhibition of M^pro^ *in vivo*. PF-00835231 suppressed HIV-1 and HCV proteases with IC_50_ values above 10 μM, which are approximately 1000-fold higher than those of coronavirus M^pro^. PF-00835231 also suppressed rhinovirus HRV3C protease with an IC_50_ of approximately 2.79 μM (Barazorda-Ccahuana et al., 2021; Liu et al., 2021). Therefore, PF-00835231 can produce a strong effect and broad-spectrum activity to suppress coronavirus M^pro^.

Interestingly, PF-07304814 also suppressed SARS-CoV-2 M^pro^ with a Ki of 174 nM, which was 644-fold higher than that of PF-00835231 (0.27 nM). PF-07304814 suppressed M^pro^ of HCoV-NL63, HCoV-HKU1, SARS-CoV-2, and MERS-CoV with IC_50_ values ranging from 0.6921 μM to 31.59 μM, which were higher than those of PF-00835231 (ranging from 8.6 nM to 383.9 nM), suggesting that the activity of PF-07304814 in suppressing M^pro^ is weaker than that of PF-00835231 (Li J, 2022). However, PF-07304814 exhibited antiviral efficacy similar to that of PF-00835231 *in vitro* (Boras et al., 2021). Therefore, PF-07304814 may itself have antiviral activity, or it may be metabolized into PF-00835231 to exert antiviral activity *in vitro*. More research on this topic is needed.

### 3.5 The mechanisms and efficacy outcomes of PF-00835231 occur simultaneously

The life cycle (replication cycle) of USA-WA1/2020 in A5491^+ACE2^ cells is approximately 4 h. Time-of-drug-addition studies have Table 1 shown that PF-00835231 inhibited USA-WA1/2020 replication in A549^+ACE2^ cells at the same time as it inhibited M^pro^, suggesting that PF-00835231 can effectively suppress SARS-CoV-2 replication in the early stage of infection (de Vries et al., 2021).

### 3.6 P-glycoprotein inhibitors increased the efficacy of PF-00835231 by suppressing its efflux from target cells

Many studies have shown that PF-00835231 is a substrate of P-gp (also called multidrug resistance protein 1 (MDR1) or ABC subfamily B member 1 (ABCB1)), which is a pump capable of promoting drug efflux from target cells (Kodan A, 2021; Taskar KS, 2022). P-gp inhibitors increase the potency of PF-00835231 *in vitro*. Specifically, PF-00835231 suppressed SARS-CoV-2 Wuhan-Hu-1, Washington strain 1, Belgium/GHB-03021/2020 infection in A549, Calu-1, Vero E6, Vero E6-enACE2 (Vero E6 kidney cells enriched with ACE2), and Vero E6-EGFP (Vero E6 cells constitutively expressing EGFP) cells with EC_50_ values ranging from 24.7 nM to 88,900 nM in the absence of P-gp inhibitors (such as CP-100356 and elacridar). In contrast, P-gp inhibitors reduced these values by 1.24- to 168.6-fold (Xi He, 2020; Boras et al., 2021). However, P-gp expression is cell type specific. P-gp was found to be expressed in only 1.77% of CD81/NK cells, 0.59% of proliferating T cells, and 0.06% of alveolar macrophages and not in other cell types (including SPP1+ macrophages, M1-like macrophages, epithelial cells, plasma cells, and neutrophils) in bronchoalveolar lavage (BAL) samples from healthy individuals. P-gp expression was also very low in BAL samples from mild and severe COVID-19 patients, suggesting that P-gp expression is extremely low in airway epithelial cells. The first and main organ to be infected by SARS-CoV-2 is the lungs along with respiratory tract cells. P-gp inhibitors (such as CP-100356) did not change the efficacy of PF-00835231 in respiratory tract cells, including HAEC, MRC-5 and A5491^+ACE2^ cells (de Vries et al., 2021). Thus, P-gp inhibitors may have little effect on the efficacy of PF-00835231 after SARS-CoV-2 infection of the respiratory epithelium. However, internally, the human body is more complicated. SARS-CoV-2 also infects multiple organs and cell types in the human body. Therefore, P-gp inhibitors may affect the efficacy of PF-00835231 *in vivo*. More research is needed.

### 3.7 Cytochrome P450 3A inhibitors enhanced the efficacy of PF-00835231 by slowing its metabolic degradation

PF-00835231 is primarily metabolized by cytochrome P450 3A4 (CYP3A4) and CYP3A5. The CYP3A inhibitor itraconazole increased the plasma concentration of PF-00835231 exposure by 2.2-fold *in vivo* (Boras et al., 2021). Indeed, CYP3A inhibitors, such as ritonavir, are strong inhibitors of CYP3A4 and are often used as effective drug boosters. A combination of ritonavir and nirmatrelvir (1:2 ratio) was approved for the treatment of COVID-19 infection by the FDA. Ritonavir enhances the plasma concentration of nirmatrelvir by 8-fold by slowing its metabolic degradation (Singh et al., 2022). Thus, CYP3A inhibitors enhanced the efficacy of PF-00835231 by slowing its metabolic degradation.

## 4 The efficacy of PF-07304814 against SARS-CoV-2 infection

### 4.1 In vitro

#### 4.1.1 PF-00835231: A broad-spectrum anti-novel coronavirus agent (EC_50_ and EC_90_ values)

PF-00835231 suppressed multiple coronavirus infections *in vitro*, including SARS-CoV-2, HCoV-229E, SARS-CoV-MA15, hCoV 229E, MERS, and SARS CoV-1, with EC_50_ values ranging from 40 nM to 5 μM. Many studies have shown that the EC90 value more accurately predicts the effective concentration of drugs *in vivo*. PF-00835231 suppressed SARS-CoV-2 replication and its induced cytopathic effects (such as ring syncytium formation and the overall number of infected foci) *in vitro*, with EC_90_ values ranging from 400 nM to 1,158 nM (Hoffman et al., 2020; Boras et al., 2021; de Vries et al., 2021). Therefore, the effective dose of PF-00835231 for inhibiting SARS-CoV-2 replication *in vivo* may be below 2 μM.

As mentioned above, the M^pro^ sequences of different coronaviruses are similar, especially at the active sites, which are 100% identical. However, the coding sequences of M^pro^ from SARS-CoV-MA15, SARS-CoV-2 C145A, SARS-CoV-2 Omicron, and SARS-CoV-2 B.1.352 (South African variant) have mutations. Interestingly, these mutation sequences are located distal to the active site. Notably, PF-00835231 still inhibited the replication of these mutant viruses in Vero E6 cells with an EC_50_ of approximately 90 nM (Boras et al., 2021). However, PF-00835231 did not inhibit the replication of other viruses in MRC-5 cells, including HRV-14, HRV-16, HIV-1 RF, HCV replicon, and HCMV RC256, suggesting that PF-00835231 may suppress only coronaviruses (de Vries et al., 2021; Sacco et al., 2022).

#### 4.1.2 The efficacy of PF-00835231 in a human respiratory epithelial organ model

SARS-CoV-2 can infect multiple organs and cell types in the human body. However, the first and main organ to be infected is the lung along with respiratory tract cells (de Freitas Santoro D, 2021). Human respiratory epithelial tissue consists of three main cell types: basal cells, secretory cells, and ciliated cells; thus, the physiological structure and function of the human respiratory epithelium cannot be replicated *in vitro*. Interestingly, human polarized airway epithelial cultures (HAECs), which contain multiple cell types (including ciliated, microfold, secretory, suprabasal, basal, and cycling basal cells), mucus and a basolateral chamber, can reproduce the typical architecture and functions of the human respiratory epithelium (Liu X, 2020; de Vries et al., 2021). This finding suggests that HAECs are one of the most physiologically relevant human respiratory epithelium organoids *in vitro* due to their characteristic polarized architecture. Interestingly, PF-00835231 strongly suppressed USA-WA1/2020-infected HAECs in a dose-dependent manner, suggesting that PF-00835231 suppresses SARS-CoV-2 infection *in vivo*.

#### 4.1.3 Synergy and additivity of PF-00835231 and remdesivir

PF-00835231 could increase remdesivir’s anti-SARS-CoV-2 efficacy because these compounds target different parts of viral replication (Boras et al., 2021). Indeed, these two drugs exhibited synergy or additivity in patient sera, suggesting that they could be combined to treat COVID-19 infection (Anirudhan V, 2021; Boras et al., 2021; de Vries et al., 2021). However, PF-00835231 did not reduce the infectious titer when used in combination with molnupiravir, suggesting that the combination of PF-00835231 and molnupiravir has no synergistic effect (Hulda R. Jonsdottir, 2022).

### 4.2 Animal model

Subcutaneous (S.C.) PF-00835231 decreased the number of multifocal pulmonary lesions and lung viral load in the BALB/c mouse model of SARS-CoV-MA15 infection. The lung viral titers were reduced by 95.98% [30 mg/kg twice daily (BID)], 99.94% (100 mg/kg BID) and 100% (300 mg/kg BID). In addition, PF-00835231 was effective in both the early and late stages of virus infection in this model (Boras et al., 2021). However, this model cannot support SARS-CoV-2 replication because ACE2 is different between mice and humans.

The adenovirus 5 human ACE2 (Ad5-hACE2) transgenic mouse model generated by SARS-CoV-2 USA-WA1/2020 infection supports virus replication *in vivo*. S.C. PF-00835231 (100 mg/kg BID) reduced lung viral titers by 96.18% in this model (Boras et al., 2021). However, the transgenic model is different from the normal model. Preclinical studies with multiple animal models are a prerequisite for the development of new drugs and subsequent clinical applications. More models, such as a mouse model of SARS-CoV-2 MA10 infection, a hamster model of SARS-CoV-2 B.1.351 (beta) infection, and a hamster model of SARS-CoV-2 B.1.617.2 (delta) infection, need to be investigated.

## 5 Pharmacokinetics of PF-07304814

### 5.1 Metabolic pathways

As mentioned above, PF-07304814 is metabolized into the active compound PF-00835231 by ALP. PF-00835231 is metabolized into four metabolites by CYP3A4 and CYP3A5. However, the structures of the metabolites have not been determined. The clearance of PF-07304814 in urine was less than 0.1% in rats, dogs, and monkeys and lower than that of PF-00835231 (7.8%, 4.8%, and 0.9%, respectively), suggesting that renal elimination is not the main clearance route of PF-07304814 and PF-0083523 (Boras et al., 2021).

### 5.2 Metabolic rate

The conversion rates of PF-07304814 into the active metabolite PF-00835231 are 68%, 81%, 78%, and 75% in rats, dogs, monkeys, and humans, respectively. Generally, an oral bioavailability (oral F) value greater than 10% is considered to show potential effectiveness after oral administration (Boras et al., 2021). However, the oral F values of PF-07304814 and PF-00835231 in rats and monkeys were very low (<2%) regardless of dose, suggesting that after oral administration, PF-07304814 and PF-00835231 have low absorption and permeability. PF-07304814 exhibited a short terminal elimination half-life (t_1/2_) in rats (0.30 h), dogs (0.5 h), monkeys (2.6 h), and humans (0.1 h), suggesting that PF-07304814 is rapidly metabolized into PF-00835231 by ALP *in vivo*. In addition, the terminal elimination t_1/2_ of PF-07304814 was lower than that of PF-0083523 [rats (0.72 h), dogs (1.5 h), monkeys (1.2 h), and humans (2 h)], suggesting that PF-07304814 is more favorable for continuous I.V. infusion than PF-0083523 (Boras et al., 2021). Taken together, these data suggest that PF-07304814 may require continuous I.V. infusion to produce antiviral activity due to its high rate of conversion to PF-00835231, low oral bioavailability, systemic clearance and short elimination half-life.

## 6 Toxicology of PF-07304814

### 6.1 In vitro

The cytotoxicity (CC_50_) values of PF-00835231 in Vero E6-enACE2, Vero E6-EGFP, and MCR5 cells were greater than 50 μM, suggesting that PF-00835231 has low toxicity. PF-07304814 and its metabolite PF-00835231 both had a negative result in the bacterial reverse mutation assay and did not induce micronuclei formation. PF-00835231 weakly inhibits CYP3A4/5 in a time-dependent manner, suggesting that the risk of drug–drug interactions (DDIs) between PF-00835231 and other drugs may be low. PF-07304814 and PF-00835231 also suppress the activity of other CYP450 enzymes and transporters, including CYP1A2, CYP2B6, CYP2C8, CYP2C9, CYP2C19, CYP2D6, CYP3A, breast cancer resistance protein (BCRP), P-gp, organic anion transporting polypeptide 1B1/3 (OATP1B1/3), organic cation transporter 1/2 (OCT1/2), organic anion transporter 1/3 (OAT1/3), and multidrug and toxin extrusion 1/2-K (MATE1/2-K). However, the IC_50_ values were all above 20 μM (Boras et al., 2021). PF-00835231 suppressed human proteases, including leukocyte elastase, chymotrypsin, pepsin, thrombin, caspase 2, and cathepsin D (CatD), with IC_50_ values above 10 μM, which are approximately 1000-fold higher than those of the coronavirus M^pro^ (Hoffman et al., 2020; Boras et al., 2021). Therefore, the off-target effect of PF-00835231 was low *in vivo*.

As mentioned above, HAECs represent a physiologically relevant organoid model. PF-00835231 did not change the tissue morphology or the epithelial layer integrity of HAECs. In addition, PF-07304814 and PF-00835231 suppressed the hERG current amplitude at concentrations greater than 300 μM, which is 125 times that of the human total maximal plasma concentration (C_max_) of PF-00835231 (2.4 µM), suggesting that the secondary pharmacology and cardiovascular toxicity of these drugs are very low. These compounds also have no effect on hemolysis, flocculation and turbidity, suggesting that they are compatible with human blood and could be suitable for I.V. administration (Boras et al., 2021; de Vries et al., 2021). Thus, PF-07304814 and PF-00835231 may have low toxicity *in vivo*.

### 6.2 Animals

**FIGURE 2:**
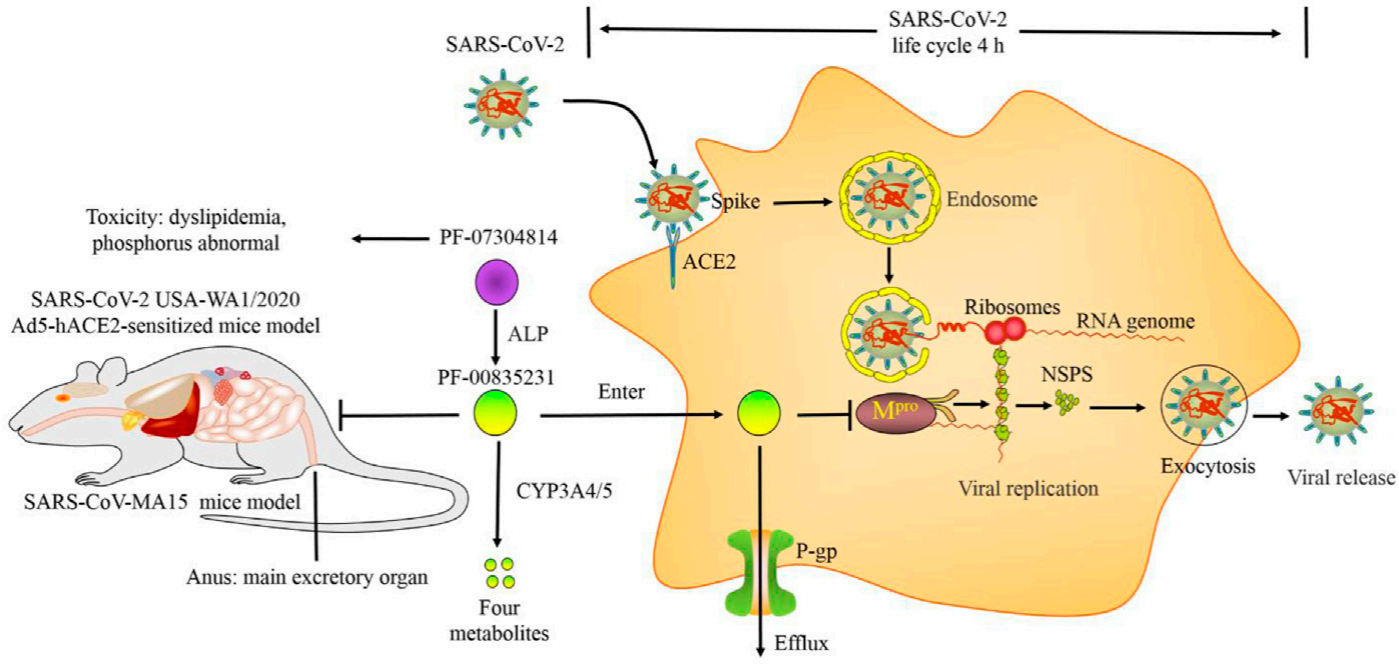
Pharmacology, pharmacokinetics and toxicology of PF-07304814.

There was no target organ or neurological toxicity in rats treated with a high dose of PF-07304814 (1,000 mg/kg, corresponding to a PF-00835231 dose of 680 mg/kg) after continuous I.V. infusion for 24 h. Due to the problem with solubility, the highest feasible and tolerable dosage of PF-00835231 is only 246 mg/kg/d in rats. There were no significant adverse effects in male rats with this dosage after continuous I.V. infusion for 4 days. There were only mild adverse reactions, including a 1.3-fold increase in cholesterol, 1.9- to 3.6-fold increases in triglycerides, and a 1.1-fold increase in phosphorus levels compared with the control (Boras et al., 2021). Taken together, these data show that PF-07304814 exhibits good tolerability, pharmacology, pharmacodynamics, pharmacokinetics, and safety (Figure 2).

## 7 Mechanism and antiviral activity of the lead structure and derivatives of PF-00835231

Lead compound PF-00835231 was patented by Pfizer (Robert Louis Hoffman et al., 2020), as were its derivatives and analogs (compounds 1–54) (Supplementary Table S1) (Robert Louis HoffmanRobert Steven KaniaJames Andrew NiemanSimon Paul PlankenGeorge Joseph Smith, 2005; Robert Steven Kania et al., 2006; Robert Louis Hoffman et al., 2020). The structures of these compounds are similar to that of PF-00835231 and form a hemithioacetal complex after the aldehyde group binds to the M^pro^ active site cysteine (Cys145) to suppress M^pro^ activity. The EC_50_ values of compounds 1-4 were on the millimolar level due to the lack of an aldehyde group (Robert Steven Kania et al., 2006), whereas the EC_50_ values of compounds 5–22 were on the micromolar level (Robert Louis HoffmanRobert Steven KaniaJames Andrew NiemanSimon Paul PlankenGeorge Joseph Smith, 2005; Hoffman et al., 2020; Robert Louis Hoffman et al., 2020), suggesting that the aldehyde group plays an important role in antiviral activity. Compounds 5–18 suppress SARS-CoV-1 infection with EC_50_ values ranging from 5 μM to 10 μM, similar to PF-00835231 (5 μM) (Robert Louis HoffmanRobert Steven KaniaJames Andrew NiemanSimon Paul PlankenGeorge Joseph Smith, 2005; Hoffman et al., 2020; Robert Louis Hoffman et al., 2020), suggesting that the antiviral activity of these compounds is similar to or slightly weaker than that of PF-00835231.

Compounds 19–22 suppress SARS-CoV-1 infection with EC_50_ values ≤5 μM, which are lower than that of PF-00835231 (5 μM), suggesting that the antiviral activities of these compounds are higher than or similar to that of PF-00835231. Notably, the EC_50_ value of compound 22 (from 0.29 to 0.35 μM) against SARS-CoV-1 infection was more than 14-fold lower than that of PF-00835231, indicating that the antiviral activity of compound 22 may be higher than that of PF-00835231 (Robert Louis HoffmanRobert Steven KaniaJames Andrew NiemanSimon Paul PlankenGeorge Joseph Smith, 2005; Hoffman et al., 2020; Robert Louis Hoffman et al., 2020). Compound 22 showed higher metabolic stability in human plasma (t_1/2_ > 240 min) than PF-00835231 (t_1/2_ = 120 min), showing that compound 22 is more favorable for use than PF-00835231 because its duration of action is longer. Compound 22 also showed low activity against endogenous glutathione (GSH) (t_1/2_ > 60 min) (Hoffman et al., 2020). GSH has a variety of functions, including as an antioxidant, modulator of DNA synthesis and repair, protector of thiol groups in proteins, stabilizer of cell membranes, and antidote for foreign organisms. GSH supplementation can be used as adjunctive therapy for many diseases, including COVID-19, hepatitis, hemolytic diseases, keratitis, cataracts and retinal diseases, suggesting that compound 22 is relatively safe (Chen D, 2022). However, compound 22 cannot be administered by I.V. due to its poor solubility (Hoffman et al., 2020). Prodrugs, nanotechnology and salt form drugs may solve this problem (Sanches BMA, 2019). Many studies are therefore needed to confirm the bioavailability, antiviral activity, metabolism and toxicity of compound 22 *in vivo*.

## 8 Future directions and challenges

More effective antiviral drugs are urgently required to combat the public health emergency caused by COVID-19. SARS-CoV-2 M^pro^ plays a key role in promoting viral replication. Importantly, M^pro^ is highly conserved among all coronaviruses. Mammalian proteases (including human proteases) do not recognize the sequence of M^pro^, suggesting that the development of highly specific M^pro^ inhibitors can reduce COVID-19 infection with fewer side effects. Indeed, many inhibitors have been found to exhibit inhibitory activity against M^pro^, including PF-07304814, PF-00835231, lopinavir/ritonavir, nirmatrelvir (also called PF-07321332), GC-376, CDI-45205, ebselen, N3, TDZD-8, and alpha-ketoamide (13b) (Agost-Beltrán L, 2022; Castillo-Garit JA, 2022). However, only oral nirmatrelvir has been approved for clinical use, while three others are in clinical trials, including FBI2001/PBI-0451 (Phase 1, NCT05011812, Pardes Biosciences Inc.), S-217622 (Phase 3, NCT05305547, NCT05363215, JPRN-jRCT2031210595, JPRN-jRCT2031210350, JPRN-jRCT2031210202, Shionogi Inc.), and PF-07304814 (Ahmad et al., 2021; Garcia-Lledo et al., 2021). Therefore, there is an urgent need to develop effective and bioavailable antiviral drugs to treat SARS-COV-2 infection. Lopinavir/ritonavir has also been used in the clinic for the treatment of COVID-19. However, lopinavir/ritonavir have high protein binding properties, and the plasma concentrations of lopinavir/ritonavir cannot reach the EC_50_ value. Lopinavir/ritonavir are also not used for the treatment of COVID-19 because they were ineffective in three randomized controlled trials (RCTs) (Tripathi N, 2022).

**FIGURE 3:**
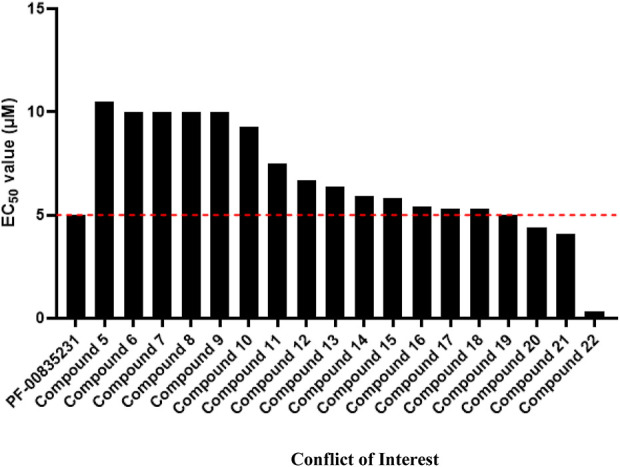
The EC_50_ value of PF-00835231 and its derivatives to suppress SARS CoV-1 229E in MRC-5 cells.

PF-07304814 was metabolized into the active compound PF-00835231 by ALP and then suppressed SARS-CoV-2 replication and infection by inhibiting M^pro^ in a preclinical trial. However, only 75% of PF-07304814 is converted to PF-00835231 in humans. Toxicity studies have shown that PF-07304814 and PF-00835231 are relatively safe but have caused dyslipidemia and phosphorus disorder in animal models. PF-00835231 derivatives bind to M^pro^ *via* a similar pathway to that of PF-00835231. The antiviral activities of PF-00835231 derivatives (compounds 5–22) to suppress SARS-CoV-1 infection are similar to or slightly weaker or higher than those of PF-00835231 *in vitro*, especially compounds 19–22 (Figure 3). However, the antiviral activity of these compounds has not been investigated or reported *in vivo*. To our knowledge, the antiviral activity of compounds 47–54 has not been investigated or reported. Further studies are needed. Notably, compound 22 exhibited the highest potency to suppress SARS-CoV-1 (14-fold greater than that of PF-00835231). Compound 22 may also have good safety and stability. However, additional studies are needed to confirm the timing of compound 22 intervention *in vivo* relative to SARS-CoV-2 infection, COVID-19 disease stage, bioavailability, antiviral activity, metabolism and toxicity. Indeed, the clinical application of compound 22 is limited due to its low solubility, but improving the solubility of compound 22 may be the key to the development of new drugs.

The Phase 1 trial of PF-07304814 as an I.V. drug was performed in hospitalized participants with COVID-19 (*N* = 26) and healthy participants (*N* = 16), and its safety, tolerability, and pharmacokinetics were evaluated (ClinicalTrials.gov Identifier: NCT04535167, NCT04627532). However, the results were not released. The Phase 2/3 trial of PF-07304814 from the ACTIV-3 trial was evaluated (NCT04501978). However, this trial was suspended. According to the news reported on their website, Pfizer initiated a Phase 2/3 study of PF-07304814 in the third quarter of 2021 (Ltd, 2021b), and the first participants were given PF-07304814 or placebo in September 2021 (Ltd, 2021c). However, this global clinical development project was discontinued for undisclosed reasons in February 2022 (Ltd, 2021a). Despite this, it is still worth studying to find more effective compounds, especially the PF-00835231 derivatives, such as compounds 19–22 and 47–54. The combination of PF-07404814 with other antiviral agents, especially those targeting different stages of the viral replication cycle, such as remdesivir, is a promising strategy to inhibit viral replication. Only by staying true to our original aspiration and persevering in research can we cope with and overcome more public health events.

SARS-CoV-2 enters the host cell by binding to the host ACE2 receptor. M^pro^ promotes the replication of SARS-CoV-2 by cleaving the polyprotein to nsps. A total of 75% of PF-07304814 is converted to PF-00835231 by ALP in humans. PF-00835231 inhibits SARS-CoV-2 replication by binding to M^pro^ and suppressing its activity. PF-00835231 suppressed the SARS-CoV-2 viral load and titers in a SARS-CoV-MA15 mouse model and SARS-CoV-2 Ad5-hACE2-sensitized mouse model. PF-07304814 exhibited a short terminal elimination t_1/2_ (0.1 h) and high PPB (81.6%) in the plasma of humans, which were lower and higher than those of PF-0083523 (2 h and 55.1%), respectively. PF-00835231 is metabolized by CYP3A4/5. P-gp promotes PF-0083523 efflux from cells; however, P-gp expression is cell type specific and extremely low in airway epithelial cells. PF-0083523 is mainly excreted in feces. Itraconazole increased the plasma nirmatrelvir concentration by approximately 2-fold by suppressing PF-00835231 metabolism. However, PF-00835231 causes dyslipidemia and phosphorus disorder.
