# Supplementary material for: Review of preclinical data of PF-07304814 and its active metabolite derivatives against SARS-CoV-2 infection
Source: Front Pharmacol. 2022 Nov 11;13:1035969. doi: 10.3389/fphar.2022.1035969 (PMC9691842; doi:10.3389/fphar.2022.1035969)
Supplement: Supplementary file 1 [file Table1.pdf]

Table S1. Structures and antiviral activities of PF-00835231 and its derivatives suppressing SARS CoV-1 229E in MRC-5 cells. Lead compound PF-00835231 was patented by Pfizer. The antiviral activities of compounds 1-4 and 23-46 were weaker than that of PF-00835231, while compounds 5-18 were similar to or slightly weaker than that of PF-00835231. The antiviral activities of compounds 19-22 are higher than or similar to that of PF-00835231, while compounds 47-54 have not been investigated or reported.

Compound	Structure	Antiviral activity	Reference(s)
PF-00835231	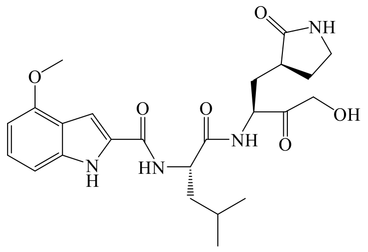	IC_{50} (M^{pro}): 0.27 nM-8 nM; EC_{50}: 5 µM	(Boras et al., 2021)
Lead compound	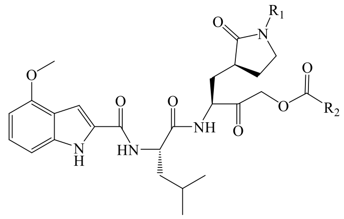	Not investigated or reported	(Robert Louis Hoffman et al., 2020)
Compound 1	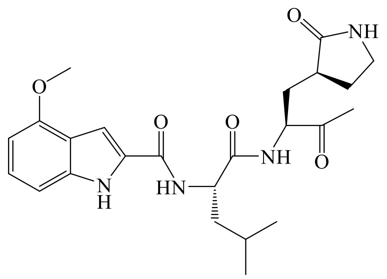	EC_{50}: 91.5 mM	(Robert Steven Kania et al., 2006)

Compound 2	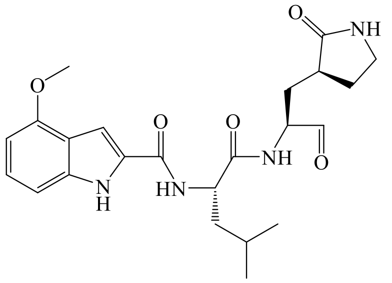	EC_{50}: 1.4 mM	(Robert Steven Kania et al., 2006)
Compound 3	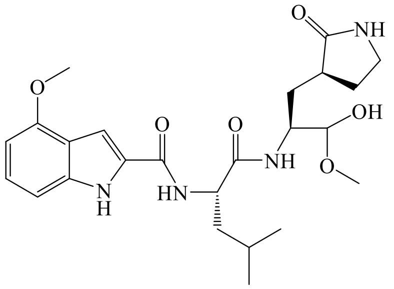	EC_{50}: 0.8 mM	(Robert Steven Kania et al., 2006)
Compound 4	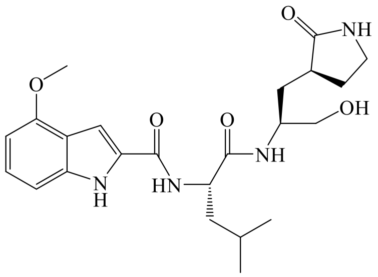	EC_{50} >100 mM	(Robert Steven Kania et al., 2006)
Compound 5	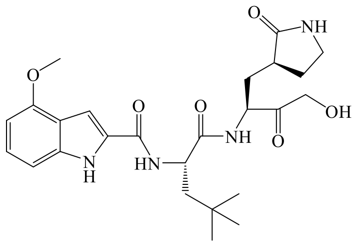	IC_{50} (M^{pro}): 7 nM or 23 nM; EC_{50}: 10 μM or 11 μM	(Robert Louis HoffmanRobert Steven KaniaJames Andrew NiemanSimon Paul PlankenGeorge Joseph Smith, 2005; Hoffman et al., 2020)

Compound 6	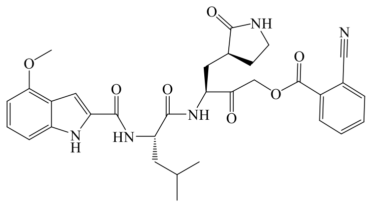	IC_{50} (M^{pro}): 17 nM; EC_{50}: 10 μM	(Robert Louis HoffmanRobert Steven KaniaJames Andrew NiemanSimon Paul PlankenGeorge Joseph Smith, 2005; Hoffman et al., 2020; Robert Louis Hoffman et al., 2020)
Compound 7	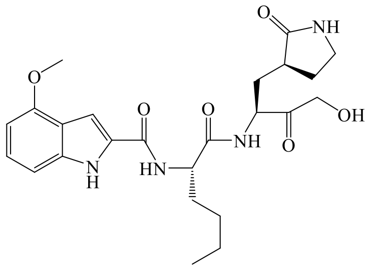	IC_{50} (M^{pro}): 20 nM; EC_{50}: 10 μM	(Robert Louis HoffmanRobert Steven KaniaJames Andrew NiemanSimon Paul PlankenGeorge Joseph Smith, 2005; Hoffman et al., 2020)
Compound 8	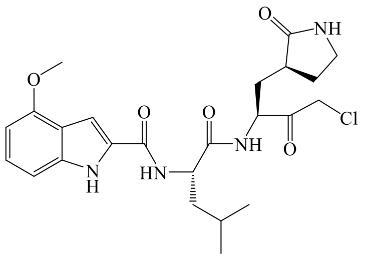	EC_{50}: 10 μM	(Robert Louis HoffmanRobert Steven KaniaJames Andrew

			NiemanSimon Paul PlankenGeorge Joseph Smith, 2005)
Compound 9	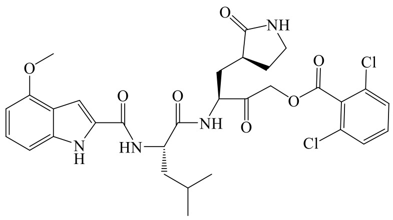	EC_{50}: 10 μM	(Robert Louis HoffmanRobert Steven KaniaJames Andrew NiemanSimon Paul PlankenGeorge Joseph Smith, 2005)
Compound 10	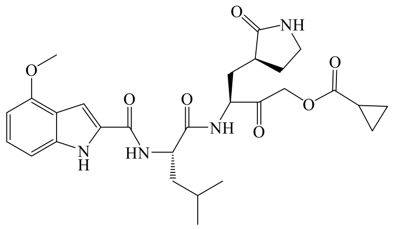	IC_{50} (M^{pro}): 180 nM or 182 nM; EC_{50}: 9.3 μM	(Robert Louis HoffmanRobert Steven KaniaJames Andrew NiemanSimon Paul PlankenGeorge Joseph Smith, 2005; Hoffman et al., 2020)

Compound 11	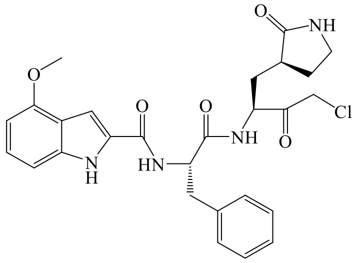	EC_{50}: 7.5 μM	(Robert Louis HoffmanRobert Steven KaniaJames Andrew NiemanSimon Paul PlankenGeorge Joseph Smith, 2005)
Compound 12	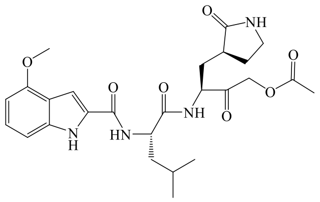	IC_{50} (M^{pro}): 220 nM; EC_{50}: 6.7 μM	(Robert Louis HoffmanRobert Steven KaniaJames Andrew NiemanSimon Paul PlankenGeorge Joseph Smith, 2005; Hoffman et al., 2020; Robert Louis Hoffman et al., 2020)
Compound 13	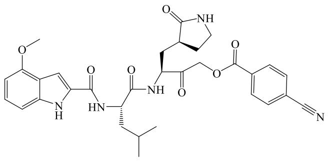	IC_{50} (M^{pro}): 53 nM; EC_{50}: 6.4 μM	(Robert Louis HoffmanRobert Steven KaniaJames Andrew NiemanSimon

			Paul PlankenGeorge Joseph Smith, 2005; Hoffman et al., 2020; Robert Louis Hoffman et al., 2020)
Compound 14	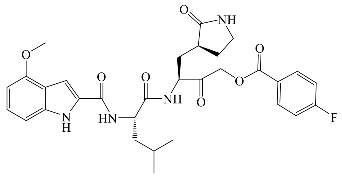	IC_{50} (M^{pro}): 82 nM; EC_{50}: 5.9 μM	(Robert Louis HoffmanRobert Steven KaniaJames Andrew NiemanSimon Paul PlankenGeorge Joseph Smith, 2005; Hoffman et al., 2020; Robert Louis Hoffman et al., 2020)
Compound 15	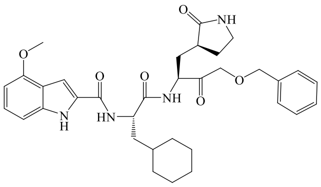	IC_{50} (M^{pro}): 133 nM; EC_{50}: 5.8 μM	(Robert Louis HoffmanRobert Steven KaniaJames Andrew NiemanSimon Paul PlankenGeorge

			Joseph Smith, 2005)
Compound 16	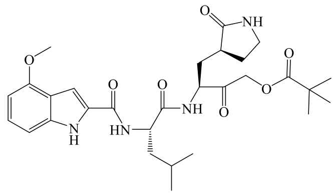	IC_{50} (M^{pro}): 230 nM; EC_{50}: 5.4 μM	(Robert Louis HoffmanRobert Steven KaniaJames Andrew NiemanSimon Paul PlankenGeorge Joseph Smith, 2005; Hoffman et al., 2020; Robert Louis Hoffman et al., 2020)
Compound 17	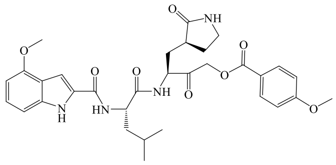	IC_{50} (M^{pro}): 79 nM; EC_{50}: 5.3 μM	(Robert Louis HoffmanRobert Steven KaniaJames Andrew NiemanSimon Paul PlankenGeorge Joseph Smith, 2005; Hoffman et al., 2020; Robert Louis Hoffman et al., 2020)

Compound 18	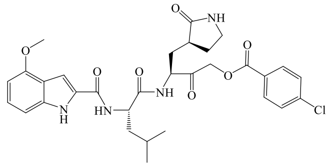	IC_{50} (M^{pro}): 97 nM; EC_{50}: 5.3 μM	(Robert Louis HoffmanRobert Steven KaniaJames Andrew NiemanSimon Paul PlankenGeorge Joseph Smith, 2005; Hoffman et al., 2020; Robert Louis Hoffman et al., 2020)
Compound 19	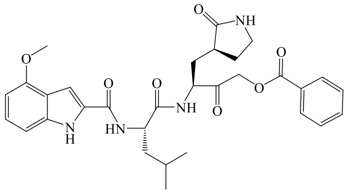	IC_{50} (M^{pro}): 86 nM; EC_{50}: 5.0 μM	(Robert Louis HoffmanRobert Steven KaniaJames Andrew NiemanSimon Paul PlankenGeorge Joseph Smith, 2005; Hoffman et al., 2020; Robert Louis Hoffman et al., 2020)

Compound 20	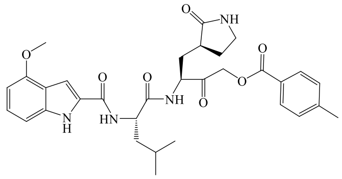	IC_{50} (M^{pro}): 87 nM; EC_{50}: 4.4 μM	(Robert Louis HoffmanRobert Steven KaniaJames Andrew NiemanSimon Paul PlankenGeorge Joseph Smith, 2005; Hoffman et al., 2020; Robert Louis Hoffman et al., 2020)
Compound 21	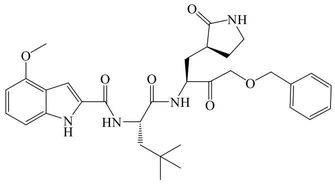	IC_{50} (M^{pro}): 32 nM; EC_{50}: 4.1 μM	(Robert Louis HoffmanRobert Steven KaniaJames Andrew NiemanSimon Paul PlankenGeorge Joseph Smith, 2005)
Compound 22	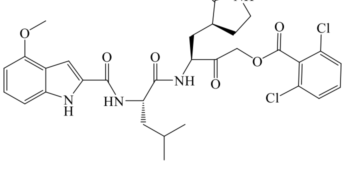	EC_{50}: 0.35 μM	(Robert Louis HoffmanRobert Steven KaniaJames Andrew NiemanSimon

			Paul PlankenGeorge Joseph Smith, 2005; Hoffman et al., 2020; Robert Louis Hoffman et al., 2020)
Compound 23	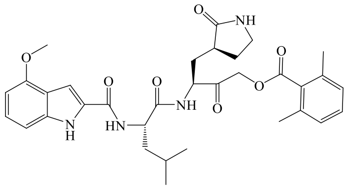	IC_{50} (M^{pro}): 74 nM or 160 nM	(Robert Louis HoffmanRobert Steven KaniaJames Andrew NiemanSimon Paul PlankenGeorge Joseph Smith, 2005; Hoffman et al., 2020; Robert Louis Hoffman et al., 2020)
Compound 24	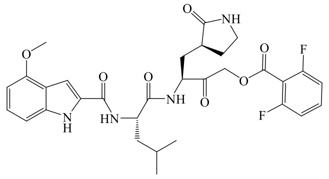	EC_{50}: 14 μM	(Robert Louis HoffmanRobert Steven KaniaJames Andrew NiemanSimon Paul PlankenGeorge

			Joseph Smith, 2005; Robert Louis Hoffman et al., 2020)
Compound 25	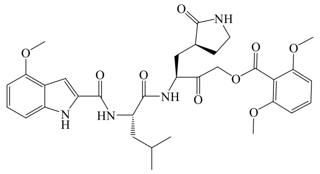	IC_{50}: 205 nM or 210 nM	(Robert Louis HoffmanRobert Steven KaniaJames Andrew NiemanSimon Paul PlankenGeorge Joseph Smith, 2005; Hoffman et al., 2020; Robert Louis Hoffman et al., 2020)
Compound 26	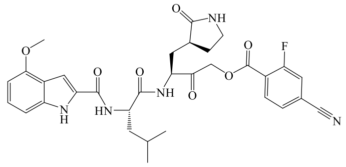	EC_{50}: 30 μM	(Robert Louis HoffmanRobert Steven KaniaJames Andrew NiemanSimon Paul PlankenGeorge Joseph Smith, 2005; Robert Louis Hoffman et al., 2020)

Compound 27	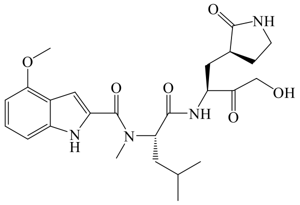	IC_{50} (M^{pro}): 83 nM or 105 nM; EC_{50}: 19 µM	(Robert Louis HoffmanRobert Steven KaniaJames Andrew NiemanSimon Paul PlankenGeorge Joseph Smith, 2005; Hoffman et al., 2020)
Compound 28	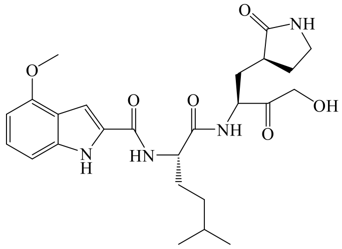	IC_{50} (M^{pro}): 34 nM, EC_{50}: 33 µM	(Robert Louis HoffmanRobert Steven KaniaJames Andrew NiemanSimon Paul PlankenGeorge Joseph Smith, 2005; Hoffman et al., 2020)
Compound 29	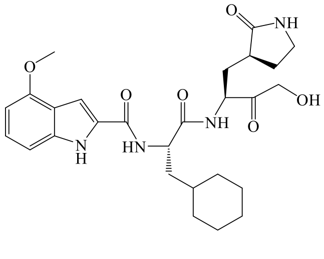	IC_{50} (M^{pro}): 25 nM or 44 nM; EC_{50}: 14 µM	(Robert Louis HoffmanRobert Steven KaniaJames Andrew NiemanSimon Paul PlankenGeorge

			Joseph Smith, 2005; Hoffman et al., 2020)
Compound 30	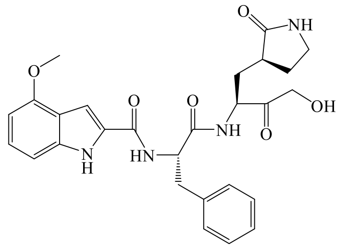	IC_{50} (M^{pro}): 103 nM; EC_{50}: 47 μM	(Robert Louis HoffmanRobert Steven KaniaJames Andrew NiemanSimon Paul PlankenGeorge Joseph Smith, 2005; Hoffman et al., 2020)
Compound 31	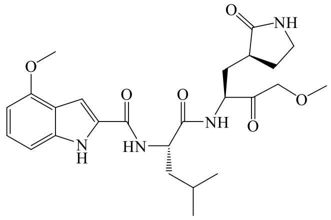	IC_{50} (M^{pro}): 51 nM; EC_{50}: 31 μM	(Robert Louis HoffmanRobert Steven KaniaJames Andrew NiemanSimon Paul PlankenGeorge Joseph Smith, 2005)
Compound 32	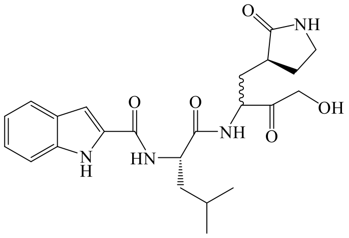	IC_{50} (M^{pro}): 38 nM; EC_{50}: 20 μM	(Robert Louis HoffmanRobert Steven KaniaJames Andrew NiemanSimon

			Paul PlankenGeorge Joseph Smith, 2005)
Compound 33	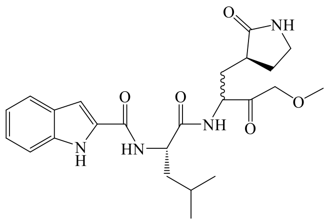	IC_{50} (M^{pro}): 131 nM; EC_{50}: 45 µM	(Robert Louis HoffmanRobert Steven KaniaJames Andrew NiemanSimon Paul PlankenGeorge Joseph Smith, 2005)
Compound 34	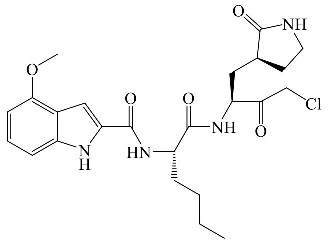	EC_{50} >42 µM	(Robert Louis HoffmanRobert Steven KaniaJames Andrew NiemanSimon Paul PlankenGeorge Joseph Smith, 2005)
Compound 35	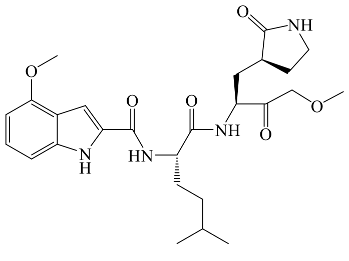	IC_{50} (M^{pro}): 36 nM; EC_{50}: 50 µM	(Robert Louis HoffmanRobert Steven KaniaJames Andrew NiemanSimon

			Paul PlankenGeorge Joseph Smith, 2005)
Compound 36	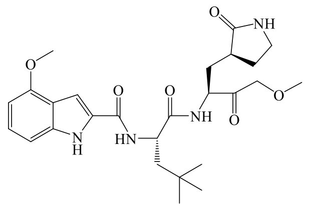	IC_{50} (M^{pro}): 54 nM; EC_{50}: 32 μM	(Robert Louis HoffmanRobert Steven KaniaJames Andrew NiemanSimon Paul PlankenGeorge Joseph Smith, 2005)
Compound 37	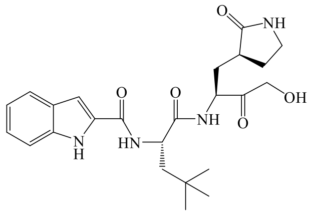	IC_{50} (M^{pro}): 20 nM; EC_{50}: 17 μM	(Robert Louis HoffmanRobert Steven KaniaJames Andrew NiemanSimon Paul PlankenGeorge Joseph Smith, 2005)
Compound 38	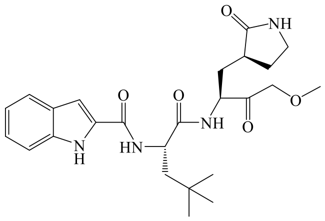	IC_{50} (M^{pro}): 105 nM; EC_{50}: 46 μM	(Robert Louis HoffmanRobert Steven KaniaJames Andrew NiemanSimon

			Paul PlankenGeorge Joseph Smith, 2005)
Compound 39	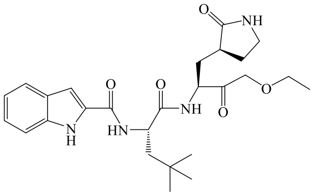	IC_{50} (M^{pro}): 112 nM; EC_{50}: 20 μM	(Robert Louis HoffmanRobert Steven KaniaJames Andrew NiemanSimon Paul PlankenGeorge Joseph Smith, 2005)
Compound 40	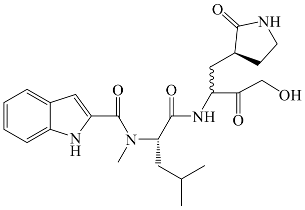	IC_{50} (M^{pro}): 152 nM; EC_{50}: 39 μM	(Robert Louis HoffmanRobert Steven KaniaJames Andrew NiemanSimon Paul PlankenGeorge Joseph Smith, 2005)
Compound 41	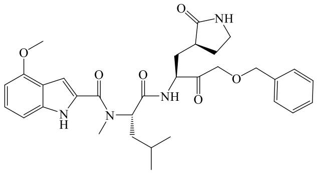	IC_{50} (M^{pro}): 927 nM; EC_{50}: 26 μM	(Robert Louis HoffmanRobert Steven KaniaJames Andrew NiemanSimon

			Paul PlankenGeorge Joseph Smith, 2005)
Compound 42	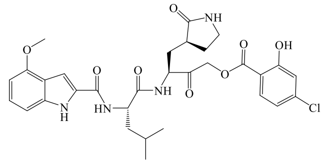	EC_{50}: 32 μM	(Robert Louis HoffmanRobert Steven KaniaJames Andrew NiemanSimon Paul PlankenGeorge Joseph Smith, 2005)
Compound 43	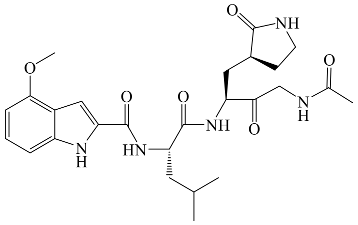	IC_{50} (M^{pro}): 2650 nM; EC_{50} >100 μM	(Robert Louis HoffmanRobert Steven KaniaJames Andrew NiemanSimon Paul PlankenGeorge Joseph Smith, 2005)
Compound 44	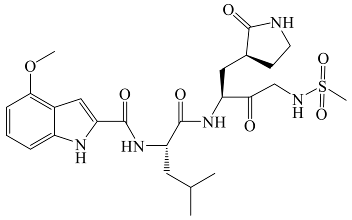	IC_{50} (M^{pro}): 107 nM; EC_{50}: 20 μM	(Robert Louis HoffmanRobert Steven KaniaJames Andrew NiemanSimon

			Paul PlankenGeorge Joseph Smith, 2005)
Compound 45	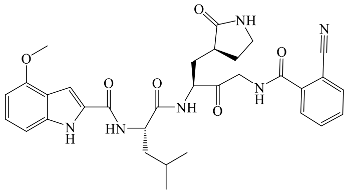	EC_{50}: >100 μM	(Robert Louis HoffmanRobert Steven KaniaJames Andrew NiemanSimon Paul PlankenGeorge Joseph Smith, 2005)
Compound 46	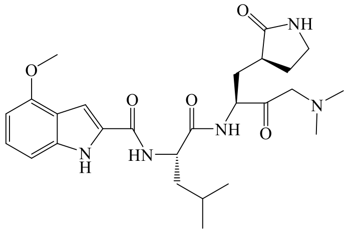	EC_{50}: >100 μM	(Robert Louis HoffmanRobert Steven KaniaJames Andrew NiemanSimon Paul PlankenGeorge Joseph Smith, 2005)
Compound 47	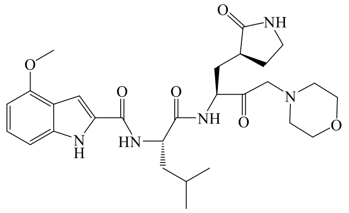	Not investigated or reported	(Robert Louis HoffmanRobert Steven KaniaJames Andrew NiemanSimon

			Paul PlankenGeorge Joseph Smith, 2005)
Compound 48	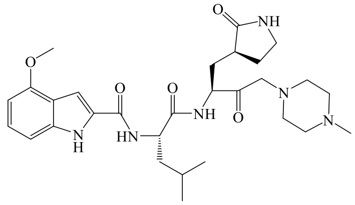	Not investigated or reported	(Robert Louis HoffmanRobert Steven KaniaJames Andrew NiemanSimon Paul PlankenGeorge Joseph Smith, 2005)
Compound 49	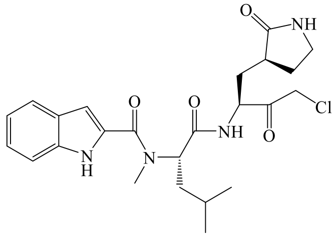	Not investigated or reported	(Robert Louis HoffmanRobert Steven KaniaJames Andrew NiemanSimon Paul PlankenGeorge Joseph Smith, 2005)
Compound 50	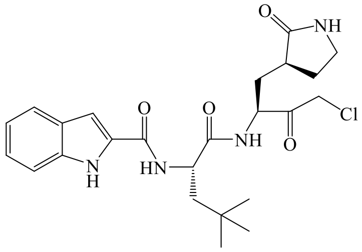	Not investigated or reported	(Robert Louis HoffmanRobert Steven KaniaJames Andrew NiemanSimon

			Paul PlankenGeorge Joseph Smith, 2005)
Compound 51	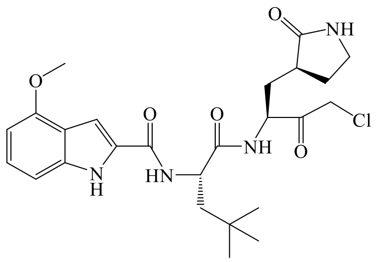	Not investigated or reported	(Robert Louis HoffmanRobert Steven KaniaJames Andrew NiemanSimon Paul PlankenGeorge Joseph Smith, 2005)
Compound 52	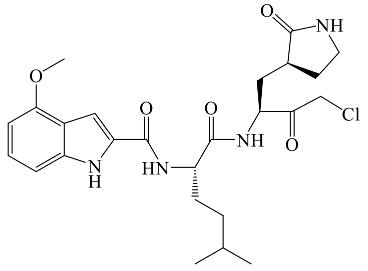	Not investigated or reported	(Robert Louis HoffmanRobert Steven KaniaJames Andrew NiemanSimon Paul PlankenGeorge Joseph Smith, 2005)
Compound 53	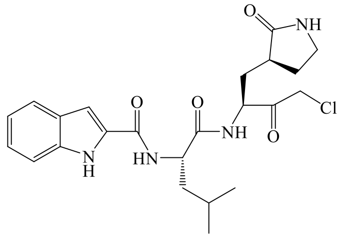	Not investigated or reported	(Robert Louis HoffmanRobert Steven KaniaJames Andrew NiemanSimon

			Paul PlankenGeorge Joseph Smith, 2005)
Compound 54	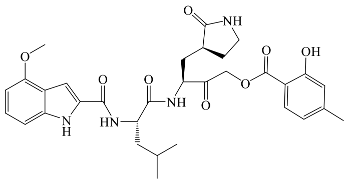	Not investigated or reported	(Robert Louis Hoffman et al., 2020)

M^{pro}: main protease, EC_{50}: concentration for 50% of maximal effect, IC_{50}: half-maximal inhibitory concentration
